# Synthesis, Crystal Structures and Luminescence Properties of Three New Cadmium 3D Coordination Polymers

**DOI:** 10.3390/molecules25112465

**Published:** 2020-05-26

**Authors:** Hai-Yan Ju, Gang Zhang, Ming Yang, De-Zheng Liu, Yong-Sheng Yang, Yan-Bo Zhang

**Affiliations:** 1 School of Chemistry and Engineering, Wuhan Textile University, 1 Textile Road, Wuhan 430073, China; juliesky2001@163.com (H.-Y.J.); prayerzg2019@163.com (G.Z.); yangming491@163.com (M.Y.); 2 Hubei Key Laboratory of Biomass Fibers and Eco-dyeing & Finishing, Wuhan Textile University, Wuhan 430073, China; 3 Hubei Key Laboratory of Power System Design and Test for Electrical Vehicle, Hubei University of Arts and Science, Xiangyang 441053, China

**Keywords:** crystal structure, 3D coordination polymer, luminescence properties

## Abstract

The new rigid planar ligand 2,5-bis(3-(pyridine-4-yl)phenyl)thiazolo[5,4-d]thiazole (BPPT) has been synthesized, which is an excellent building block for assembling coordination polymer. Under solvothermal reaction conditions, cadmium ion with BPPT in the presence of various carboxylic acids including (1,1′-biphenyl)-4,4′-dicarboxylic acid (BPDC), isophthalic acid (IP), and benzene-1,3,5-tricarboxylic acid (BTC) gave rise to three coordination complexes, viz, [Cd(BPPT)(BPDA)](BPPT)n (**1**), [Cd(BPPT) (IP)] (CH_3_OH) (**2**), and [Cd_3_(BPPT)_3_(BTC)_2_(H_2_O)_2_] (**3**). The structures of **1**, **2**, and **3** were characterized by single crystal X-ray diffraction. The IR spectra as well as thermogravimetric and luminescence properties were also investigated. Complex **1** is a two-dimensional (2D) network and further stretched to a 3D supramolecular structure through π–π stacking interaction. The complexes **2** and **3** show 3D framework. The complexes **1**, **2**, and **3** exhibited luminescence property at room temperature.

## 1. Introduction

In the last twenty years, the design and synthesis of new coordination polymers (CPs) have become an important research area for their intriguing structures and interesting properties, such as catalysis [1,2,3,4,5,6], gas storage and separation [7,8,9], photoluminescence [10,11,12], biomedical uses [13], and other applications [14,15,16]. In order to make the CPs have special structures and properties, it is necessary to synthesize new ligands [17], in which the heteroaromatic thiazolothiazole unit can be employed as a building block incorporated into organic ligands; the strong π–π stacking and overlapping of the orbitals in thiazolothiazole unit can afford high electron and hole mobilities, which are crucial properties for efficient charge transfer in optoelectronic materials [18]; and the heteroaromatic thiazolothiazole unit has also shown high luminescence properties [19]. On the other hand, the luminescence properties of coordination polymers with d 10 metal centres Cd(II) have attracted intense interest because of the potential applications of these compolexes as luminescent sensing materials [20]. Therefore, the design and synthesis of diverse structural Cd–MOFs or Cd–CPs with optical properties are highly demanded [21,22].

Here, we have selected a new ligand, namely 2,5-bis(3-(pyridine-4-yl)phenyl)thiazolo[5,4-d]thiazole (BPPT). In the present report, we used BPPT as a ligand to self-assemble with Cd(II) ion and obtained a new three-dimensional coordination polymer, namely [Cd(BPPT)(BPDA)](BPPT)n (**1**), [Cd(BPPT) (IP)] (CH_3_OH) (**2**) and [Cd_3_(BPPT)_3_(BTC)_2_(H_2_O)_2_] (**3**). The structure was characterized by single crystal X-ray diffraction. The IR spectra as well as thermogravimetric and luminescence properties were also investigated.

## 2. Results and Discussion

### 2.1. The Structure of Complex ***1***

**Figure 1:**
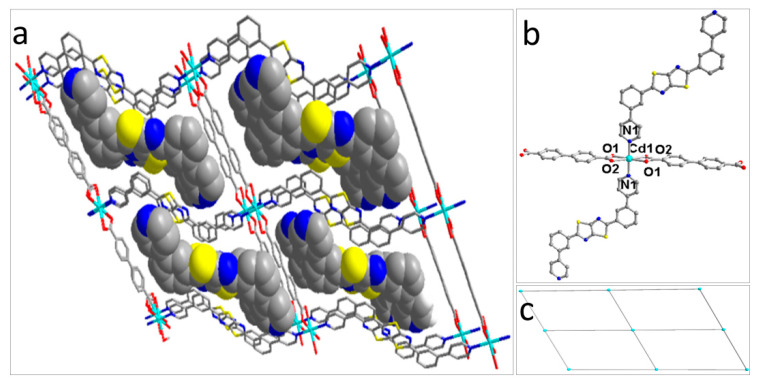
The 3D network of **1** (**a**), the coordination environment of Cd(II) in **1** (**b**), and the topological network formed in **1** (**c**).

Single-crystal X-ray diffraction indicated that complex **1** crystallizes in triclinic P-1 space group and displays a two-dimensional (2D) structure, which contains two BPPT, one deprotonated BPDA ligand, and one Cd(II) cation in the formula unit. A “Z” shape cavity is formed via the coordination interaction of Cd(II) (Cd-N and Cd-O) among BPPT and BPDA ligands, which can further propagate to an infinite 2D lamellar framework. As shown in Figure 1a, a 3D layer-type network architecture is formed owing to the presence of π–π stacking interactions between the layers. Interestingly, there is exactly one BPPT molecule in the “Z” shape cavity, and the closest distance between two parallel π-stacked BPDA molecules was around 3.56 Å. Each Cd(II) ion, adopting an octahedral geometry, is equatorially coordinated by four oxygen atoms from two BPDA ligands and two axially coordinated nitrogen atoms from BPPT (Figure 1b). The Cd-O bond lengths range over 2.303(2)–2.461(2) Å and the Cd-N bond length is 2.351(3) Å. Selected bond distances and angles are listed in Table 1. The BPDA ligands and BPPT ligands are considered as linkers to generate a 4-connected 2D network with **sql** topology (Figure 1c).

### 2.2. The Structure of Complex ***2***

**Figure 2:**
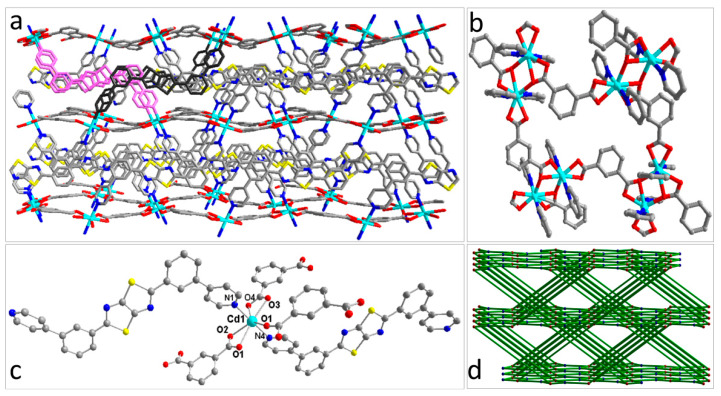
The 3D network of **2** (**a**), the 2D [Cd(IP)] n layer in **2** (**b**), the coordination environment of Cd(II) in **2** (**c**), and the topological network formed in **2** (**d**); red spheres represent Cd(II) centers, blue spheres represent the isophthalic acid (IP^−^) ligands.

The X-ray structural analysis reveals that complex **2** crystallizes in the monoclinic space group P21/c, which is a three-dimensional (3D) coordination polymers constructed from BPPT and IP ligands (Figure 2a). The structural unit is made up of one Cd (II) atom, one BPPT ligand, and one IP ligand. Cd (II) ions are bridged by IP ligands to form a 2D wavelike network along the a axis (Figure 2b). It is noteworthy that the Cd (II) is a seven coordinated ion (Figure 2c), in which five oxygen atoms from IP ligand are in equatorial plane to form a two-dimensional structure (Figure 2b). The Cd-O bond distances vary from 2.366(16) to 2.393(17) Å. The O-Cd-O bond angles are 53.3(5)°–166.5(6)°, respectively. Two pyridyl nitrogen atoms coordinate with Cd (II) in the axial direction to form a three-dimensional structure with a Cd-N bond distance of 2.317(18) Å. The N-Cd-N bond angle is 176.5(7)°. That is to say, the Cd(II) ions are assembled via bridging carboxylate oxygen atoms in a 2D plane, and these planes are further interconnected by the BPPT ligand, giving rise to a 3D framework. The closest distance between two parallel π-stacked DPPZ ligands was around 3.55 Å, that is, within the range of π−π interaction. Topological analysis through the olex, the IP ligands, and BPPT ligands are considered as linkers, then the 3D structure can be classified as a 5-connected network with (4.8^4^)(4.6^4^.8^4^.10) topology (Figure 2d).

### 2.3. The Structure of Complex ***3***

**Figure 3:**
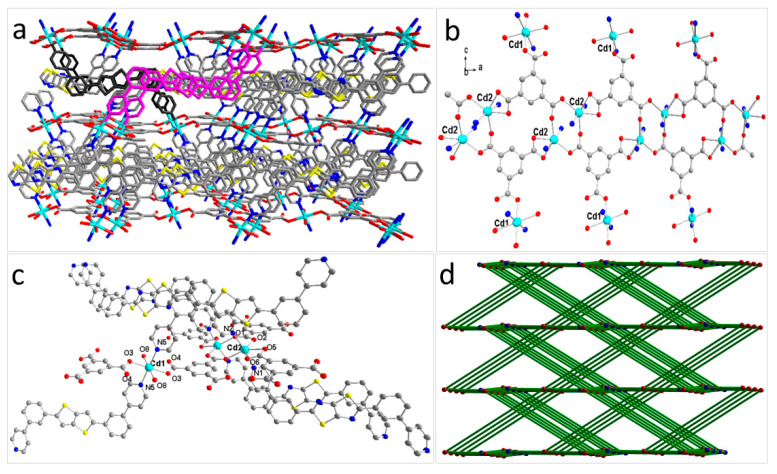
The 3D network of **3** (**a**), the 2D [Cd(BTC)] n layer in **3** (**b**), the coordination environment of Cd(II) in 3 (**c**), and the topological network formed in **3** (**d**); red spheres represent Cd(II) centers, blue spheres represent the benzene-1,3,5-tricarboxylic acid (BTC^−^) ligands.

According to the single crystal-XRD analysis, complex **3** also crystallized in the monoclinic crystal system with space group of P21/c and its asymmetric unit contains three Cd(II) ions, three BPPT ligands, two BTC ligands, and two water molecules. Complex **3** has a 3D structure (Figure 3a). The binding of Cd(II) ions to the organic BTC ligand generates the 2D layer coordination structure, one carboxylic acids group in the BTC coordinates to Cd (1) ion, and the other two carboxylic groups in the BTC coordinates to Cd (2) (Figure 3b). The 2D layer interconnected by the BPPT ligand gives rise to a 3D framework and the closest distance between the two parallel π-stacked DPTTZ ligands was around 3.57 Å. As shown in Figure 3c, the metal atom Cd (1) is six-coordinate and in an octahedral geometry coordination environment with two oxygen atoms (O3, O8) from BTC ligands, two water molecules, and two nitrogen atoms (N3, N3) from BPPT. The Cd (1)-O3 bond length is 2.253(5) Å, the Cd (1)-O8 bond length is 2.346(9) Å, and the Cd (1)-N bond length is 2.319(6) Å. The O-Cd (1)-O angles are in the range of 82.2(3)°–180.00(11)°, the N-Cd (1)-O angles are in the range of 88.7(2)°–91.3(2)°, and the N-Cd (1)-N angle is 180.0(3)°. Each Cd (2) ion is located in an octahedral coordination environment. Four oxygen atoms from two BTC ligands and two nitrogen atoms from two BPPT ligands in the axial direction, the Cd (2)-N bond distance of 2.317(18) Å, and the N-Cd (2)-N bond angle is 176.5(7)°. The Cd (2)-O bond lengths are in the range of 2.255(4) Å–2.398(5) Å. The O-Cd (2)-O angles are in the range of 55.02(16)°–146.95(17)°. The BTC ligands and BPPT ligands are considered as linkers, thus the 3D structure can be classified as a 4, 5-connected network with (4^2^.6.8^4^.9)(8.9^3^.10^2^)(4^2^.6^7^.8) topology (Figure 3d).

### 2.4. TGA

**Figure 4:**
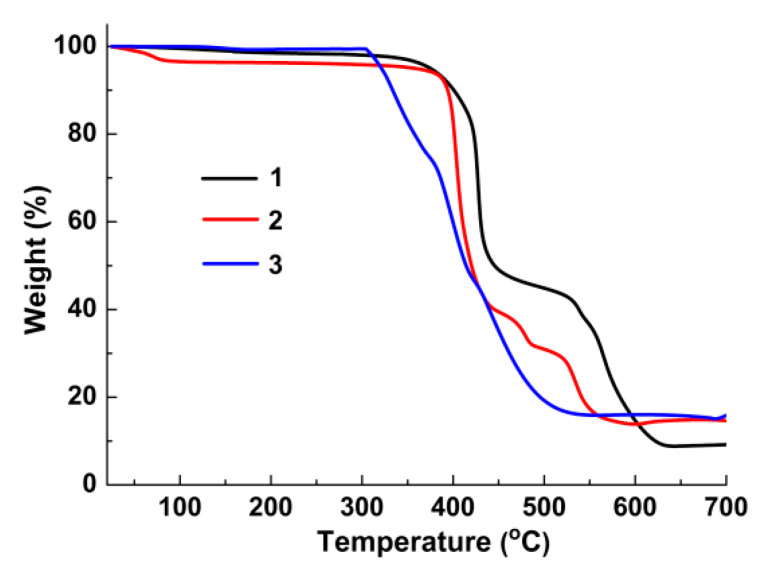
Thermal gravimetric analysis (TGA) curves of complexes **1**–**3**.

TGA was conducted to investigate the thermal stability of complexes **1**, **2**, and **3**. As shown in Figure 4, the TG curve of **1** displayed no obvious weight loss before 380 °C, the weight loss of 49.1% can be distributed to the removal of BPPT molecule in the “Z” shape cavity (calcd. 35.9%), and the framework to decompose at 380–445 °C, the remaining weight corresponds to the constitution of CdO (calcd. 11.8%, found 9.2%) at 700 °C. The TGA for complex **2** shows a weight loss of 3.8% (calculated 4.1%) before 75 °C, which is associated with the loss of one free methanol molecule. When the temperature reaches 400 °C, the framework of **2** begins to break down gradually. The weight loss of 85.5% (calculated 81.1%) at 700 °C can be ascribed to the decomposition of organic matters in complex **2**. According to the TGA results, complex **3** exhibited no obvious weight loss before 310 °C. After that temperature, the framework begins to decompose. The weight loss of 84.1% (calculated 79.4%) at 700 °C can be ascribed to the decomposition of organic matters in complex **3**.

### 2.5. IR Analysis

**Figure 5:**
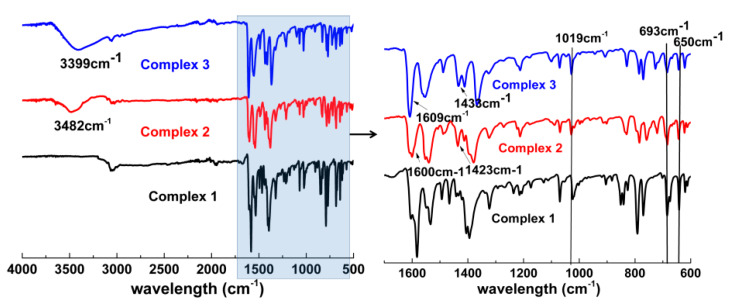
Comparison of FT-IR spectrum of complexes **1**, **2**, and **3**.

In the IR spectra of complexes **1**, **2**, and **3** (Figure 5), the broad bands at about 3482 cm^−1^ for **2** and 3399cm^−1^ for **3** are assigned to the presence of methanol (**2**) water molecules (**3**). All three complexes displayed similar FT-IR spectra with only a little variation in the peak position with regards to the -C=N (1019 cm^−1^) and -C-S bonds (693 cm^−1^ and 650 cm^−1^) of the BPPT [23]. The characteristic peaks of the deprotonated –COO– symmetry band and the asymmetric band appeared at 1600 cm^−1^ and 1423 cm^−1^ for **2** and 1609 cm^−1^ and 1438 cm^−1^ for **3**, respectively.

### 2.6. UV/Vis Analysis

**Figure 6:**
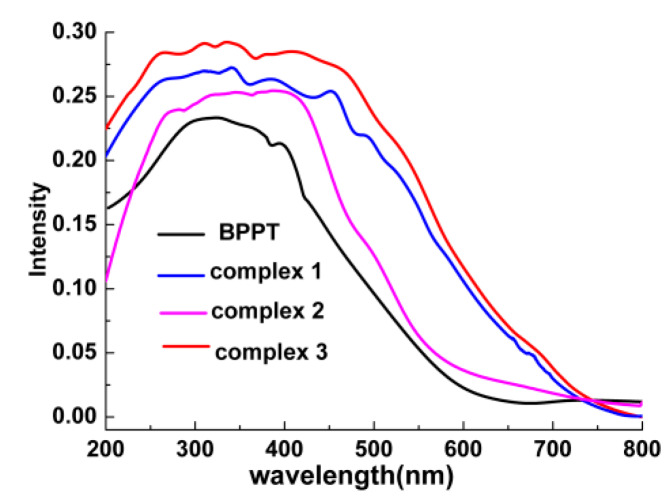
UV/vis spectrogram of 2, 5-bis(3-(pyridine-4-yl)phenyl)thiazolo[5,4-d]thiazole (BPPT) as well as complexes **1**, **2**, and **3** in the solid state.

From the spectrum of UV/vis (Figure 6), the maximum absorption of BPPT, **1**, **2**, and **3** occurred at 330 nm, 340 nm, 330 nm, and 344 nm, respectively. The bands could be assigned to characteristic π–π* transitions centered on BPPT. Compared with the free ligand BPPT, the absorption peaks of **1**, **2**, and **3** have slightly red-shift, probably attributed to the coordination of the ligands to the Cd ion, which effectively increases the conjugated extent of the complexes [24].

### 2.7. Luminescence Properties

**Figure 7:**
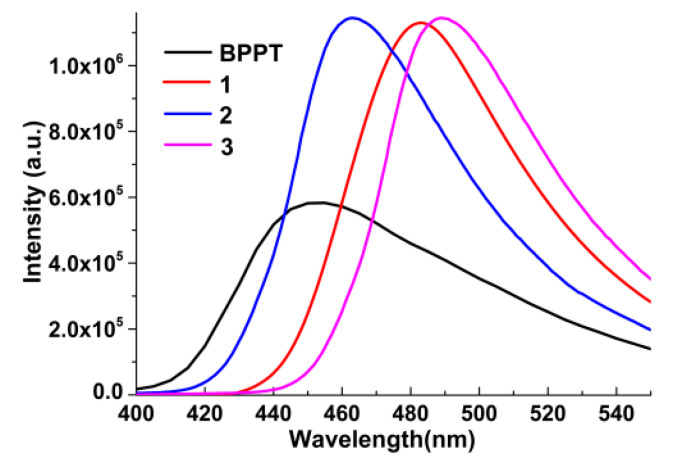
The emission spectra of BPPT, **1**, **2**, and **3** in the solid state at room temperature.

The luminescence properties of complexes **1**, **2**, and **3** were investigated in the solid state at room temperature. The emission spectra are shown in Figure 7, and the complexes **1**, **2**, and **3** exhibit luminescence properties, with an emission at 483 nm (excited at 340 nm), 463 nm (excited at 330 nm), and 489 nm (excited at 344 nm), respectively. To understand the nature of the emission spectra, the luminescence property of free BPPT ligand was also investigated in the solid state under the same experimental conditions. The BPPT ligand exhibits one emission band at 455 nm upon excitation at 330 nm. This suggests that the emission of **1**, **2**, and **3** originated from the π–π* electronic transition of the ligand [25]. By comparing the emission spectra of complexes **1**, **2**, and **3** and the free BPPT ligand, we can conclude that the enhancement of luminescence in **1**, **2**, and **3** may be attributed to the ligation of ligand to the metal center, which effectively increases the rigidity and reduces the loss of energy by radiation less decay [26,27]. A detailed spectroscopic study of a possible structure-related luminescent is underway.

## 3. Experiment

### 3.1. Material and Physical Measurements

**Scheme 1:**
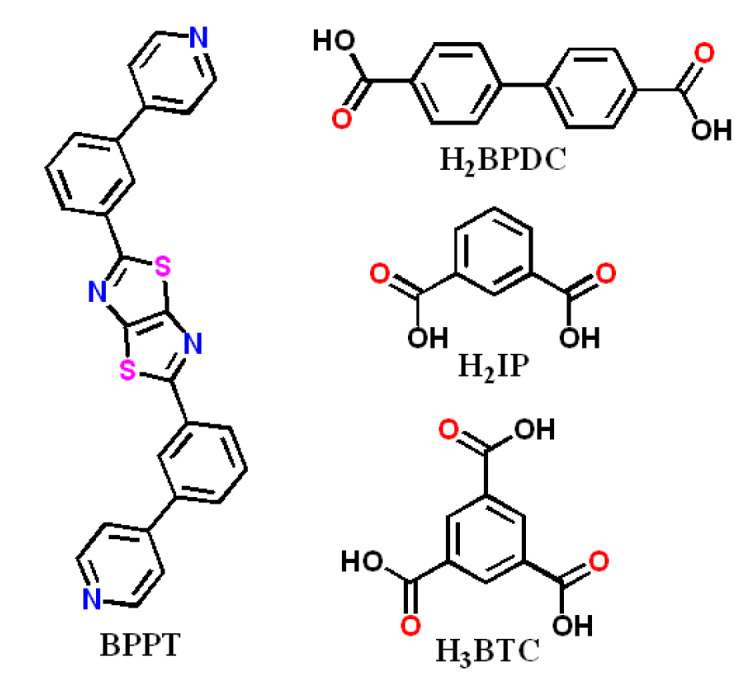
The structures of ligands used in the syntheses of the coordination polymers. BPPT, 2, 5-bis(3-(pyridine-4-yl)phenyl)thiazolo[5,4-d]thiazole; BPDC, (1,1′-biphenyl)-4,4′-dicarboxylic acid; IP, isophthalic acid; BTC, benzene-1,3,5-tricarboxylic acid.

The organic ligand BPPT was synthesized by modifying the reported procedure using 3-(pyridine-4-yl)benzaldehyde [28]. 3-Bromobenzaldehyde, 4-Pyridineboronic acid, dithiooxamide (Macklin Biochemical Co., Ltd., Shanghai, China) and other reagents (Fuyu Chemical Co., Ltd., Tianjin, China), were used without further purification. The structures of ligands used in the syntheses of the coordination polymers are shown in Scheme 1. Elemental analyses for C, H, and N were performed on a Perkin-Elmer 240C Elemental Analyzer (PerkinElmer, Waltham, MA, USA) at the analysis center of Nanjing University. FT-IR (Fourier transform infrared) spectra were recorded in the range of 400–4000 cm^−1^ on a Bruker Vector 22 FT-IR spectrophotometer (Bruker, Karlsruhe, Germany) using KBr pellets. Powder X-ray diffraction (PXRD) measurements were carried out on a Bruker D_8_ Advance X-ray diffractometer (Bruker, Karlsruhe, Germany) using Cu-Kα radiation (λ = 1.5418 Å). Thermal gravimetric analyses (TGAs) were taken on a Mettler–Toledo thermal analyzer (Mettler–Toledo, Greifensee, Switzerland) under an N2 atmosphere with a heating rate of 10 °C·min^−1^. The luminescence spectra were measured on a Perkin Elmer LS-55 fluorescence spectrophotometer (PerkinElmer, Waltham, MA, USA).

### 3.2. Synthesis of 3-(Pyridine-4-yl) Benzaldehyde

3-Bromobenzaldehyde (1.98 g, 10.72 mmol) was dissolved in 20 mL of 1, 4-dioxane and 20 mL of water. Then, 4-Pyridineboronic acid (1.26 g, 10.25 mmol) was added and dissolved, followed by the addition of K_2_CO_3_ (4.2 g, 17.36 mmol) and Pd(PPh_3_)_4_ (290 mg, 0.25 mmol). The mixture was refluxed under the protection of N_2_ gas and stirred for 72 h at 80 °C. After cooling to room temperature, the solvent was removed under reduced pressure to obtain the white solid. The residue was purified by silica gel column chromatography using petroleum ether and ethyl acetate (*v*/*v*, 1/1) as eluent to afford 3-(pyridine-4-yl) benzaldehyde. (Yield, 81.0%). ^1^H NMR (400 MHz, CDCl_3_) δ 10.16–10.08 (m, 1H), 8.72 (d, *J* = 5.5 Hz, 2H), 8.16 (s, 1H), 7.94 (dd, *J* = 19.5, 7.7 Hz, 2H), 7.69 (t, *J* = 7.7 Hz, 1H), 7.55 (t, *J* = 6.5 Hz, 2H). IR (KBr pellet cm^−1^): 3029(m), 2826(m), 2730(m), 1949(s), 1690(s), 1601(s), 1583(s), 1413(s), 1302(s), 1189(s), 789(s), 654(m), 613(m), 579(m).

### 3.3. Synthesis of 2,5-Bis(3-(pyridine-4-yl)phenyl)thiazolo[5,4-d]thiazole (BPPT)

A mixture of 3-(pyridine-4-yl)benzaldehyde (1.83 g, 10.0 mmol) and dithiooxamide (0.60 g, 5.0 mmol) in DMF (20 mL) was stirred at 150 °C for 24 h. The residue was purified by silica gel column chromatography using dichloromethane (DCM) as eluent to afford L (yield, 81.0%). ^1^H NMR (400 MHz, CHCl_3_): ^1^H NMR (400 MHz, CDCl_3_) δ 8.73 (dd, *J* = 4.5, 1.6 Hz, 2H), 8.32 (t, *J* = 1.7 Hz, 1H), 8.05 (ddd, *J* = 13.4, 7.8, 6.4 Hz, 1H), 7.79–7.70 (m, 1H), 7.67–7.57 (m, 3H), 2.95 (dd, *J* = 28.8, 22.7 Hz, 1H). IR(KBr pellet cm^−1^): 3033(m), 1661(w), 1597(s), 1533(m), 1469(m), 1286(m), 1222(m), 1020(m), 791(s), 673(s), 609(s).

### 3.4. Synthesis of [Cd(BPPT)(BPDA)](BPPT)n *(**1**)*

A mixture of BPPT (15.0 mg, 0.03 mmol), H_2_BPDA (7.2 mg, 0.03mmol), Cd(ClO_4_)_2_ (2.1 mg, 0.06 mmol), and DMF/water (15 mL, 1:1 *v*/*v*) was placed in a 20 mL Teflon-lined stainless steel autoclave. The mixture was heated under autogenous pressure at 433 K for 72 h and then cooled to room temperature. Yellow block-shaped crystals were collected by filtration, washed with H_2_O, and dried in air (yield 67%, based on BPPT). Analysis calculated for C_66_H_40_CdN_8_O_4_S_4_: C 63.43, H 3.23, N 8.97%; found: C 63.40, H 3.27, N 8.96%. IR (KBr pellet cm^−1^): 3043(w), 1569(s), 1524(s), 1451(m), 1386(s), 1323(m), 1203(w), 838(m), 773(s), 691(m), 650(m), 627(m).

### 3.5. Synthesis of [Cd(BPPT) (IP)] (CH_3_OH) *(**2**)*

A mixture of BPPT (15.0 mg, 0.03 mmol), H_2_IP (4.8 mg, 0.03mmol), Cd(NO_3_)_2_ (2.1 mg, 0.06 mmol), and methanol/water (15 mL, 1:1 *v*/*v*) was placed in a 20 mL Teflon-lined stainless steel autoclave. The mixture was heated under autogenous pressure at 433 K for 72 h and then cooled to room temperature. Yellow block-shaped crystals were collected by filtration, washed with H_2_O, and dried in air (yield 67%, based on BPPT). Analysis calculated for C_35_H_26_CdN_4_O_6_S_2_: C 54.23, H 3.38, N 7.23%; found: C 54.18, H 3.42, N 7.24%. IR (KBr pellet cm^−1^): 3482(m), 3024(w), 1600(s), 1587(s), 1551(s), 1435(m), 1423(m), 1377(s), 1203(m), 1057(m), 1020(w), 755(m), 690(m), 673(m).

### 3.6. Synthesis of [Cd_3_(BPPT)_3_(BTC)_2_(H_2_O)_2_] *(**3**)*

A mixture of BPPT (15.0 mg, 0.03 mmol), H_3_BTC (6.3 mg, 0.03 mmol), Cd(NO_3_)_2_ (21.0 mg, 0.06 mmol), and ethanol/water (15 mL, 1:1 *v*/*v*) was placed in a 20 mL Teflon-lined stainless steel autoclave. The mixture was heated under autogenous pressure at 433 K for 72 h and then cooled to room temperature. Yellow block-shaped crystals were collected by filtration, washed with H_2_O, and dried in air (yield 67%, based on BPPT). Analysis calculated for C_96_H_61_Cd_3_N_12_O_14_S_6_: C 53.98, H 2.88, N 7.87%; found: C 53.95, H 2.95, N 7.83%. IR (KBr pellet cm^−1^): 3399(w), 1609(s), 1552(s), 1488(w), 1433(m), 1414(m), 1367(m), 1213(w), 1020(w), 791(w), 690(m), 682(w), 618(w).

### 3.7. X-Ray Crystallography

Diffraction data for the complex were collected at 293 (2) K, with a Bruker Smart 1000 CCD diffractometer using Mo-Kα radiation (λ = 0.71073 Å) with the ω-2θ scan technique. An empirical absorption correction was applied to raw intensities [29]. The structure was solved by direct methods (SHELX-97) and refined with full-matrix least-squares technique on F_2_ using the SHELX-97 [30]. The hydrogen atoms were added theoretically, and riding on the concerned atoms and refined with fixed thermal factors. Crystal data, data collection, and structure refinement details are summarized in Table 1. Suitable single crystals of **1**, **2**, and **3** were selected and mounted in air onto thin glass fibers. In these structures, H atoms bonded to C atoms were treated as riding in geometrically idealized positions, with C-H = 0.93 Å and *U*_iso_ (H) = 1.2*U* eq (C), and the selected bond lengths and angles with their estimated standard deviations are listed in Table 2.

## 4. Conclusions

The new rigid planar ligand BPPT has been synthesized, by introducing different rigid carboxylic acids BPDC, IP, and BTC as auxiliary ligands; three Cd(II) CPs have been synthesized successfully under solvothermal conditions. The complexes **1**, **2**, and **3** show a 3D framework and have good thermal stability. In addition, complexes **1**, **2**, and **3** exhibit luminescence properties. We hope that this new BPPT ligand can obtain more novel structures. Studies toward the preparation of new CPs or MOFs, especially further studies of luminescence properties including the three complexes, are underway.

## Figures and Tables

**Table 1:** Crystal data and structure refinement for **1**, **2**, and **3**.

Complex	1	2	3
Empirical formula	C_66_H_40_CdN_8_O4S_4_	C_35_H_26_CdN_4_O_6_S_2_	C_96_H_61_Cd_3_N_12_O_14_S_6_
Formula weight	1249.71	769.08	2113.01
Temperature	293(2) K	293(2) K	293(2) K
Wavelength	1.54178 A	1.54178 A	1.54178 A
Crystal system, space group	Triclinic, P-1	Monoclinic, P21/c	Triclinic, P-1
Unit cell dimensions	a = 5.4580(4) Å α = 76.509(5) deg.	a = 12.7996 (2) Å α = 90 deg.	a = 10.4278(4) Å α = 88.931(4) deg.
	b = 15.3295(9) Å β = 85.570(6) deg.	b = 17.9295(3) Å β = 96.4141(14) deg.	b = 12.5607(7) Å β = 89.037(4) deg.
	c = 16.3168(11) Å γ = 82.198(5) deg.	c = 14.5012(2) Å γ = 90 deg.	c = 18.2058(8) Å γ = 83.109(4) deg.
Volume	1313.77(15) Å^3^	3307.07(8) Å^3^	2366.71(19) Å^3^
Z, Calculated density	1, 1.580 Mg/m^3^	4, 1.559 Mg/m^3^	1, 1.483 Mg/m^3^
Absorption coefficient	5.324 mm^−1^	6.908 mm^−1^	7.147 mm^−1^
F(000)	636	1544	1057
Crystal size	0.3 × 0.2 × 0.1 mm	0.3 × 0.25 × 0.2 mm	0.3 × 0.2 × 0.14 mm
Theta range for data collection	3.60 to 70.95 deg.	3.47 to 70.99 deg.	4.26 to 71.09 deg.
Limiting indices	−5 ≤ h ≤ 6, −18 ≤ k ≤ 18, −19 ≤ l ≤ 19	−15 ≤ h ≤ 11, −21 ≤ k ≤ 21, −16 ≤ l ≤ 17	−12 ≤ h ≤ 8, −15 ≤ k ≤ 15, −22 ≤ l ≤ 22
Reflections collected/unique	8494/5078 [R(int) = 0.0331]	12555/6390 [R(int) = 0.0204]	16199/9166 [R(int) = 0.0426]
Completeness to theta	97.5%	98.1%	97.3%
Absorption correction	Semi-empirical from equivalents	Semi-empirical from equivalents	Semi-empirical from equivalents
Refinement method	Full-matrix least-squares on F^2	Full-matrix least-squares on F^2	Full-matrix least-squares on F^2
Data/restraints/parameters	4951/0/376	6268/6/433	8917/0/592
Goodness-of-fit on F^2	0.938	1.061	1.047
Final R indices [I > 2sigma(I)]	R1 = 0.0407, wR2 = 0.1112	R1 = 0.0388, wR2 = 0.1085	R1 = 0.0725, wR2 = 0.2180
R indices (all data)	R1 = 0.0473, wR2 = 0.1167	R1 = 0.0416, wR2 = 0.1107	R1 = 0.0797, wR2 = 0.2300
Largest diff. peak and hole	0.488 and −0.819	2.027 and −1.389 e.A^−3^	3.271 and −1.542 e.A^−3^

**Table 2:** Selected bond lengths (Å) and bond angles (°) of complex **1**, **2**, and **3**.

**Complex 1**					
Bond	Dist.	Bond	Dist.	Bond	Dist.
Cd(1)-O(1)	2.303(2)	Cd(1)-N(1)	2.351(3)	Cd(1)-O(2)	2.461(2)
Angle	(°)	Angle	(°)	Angle	(°)
O(1)#2-Cd(1)-O(1)	180.00(12)	O(1)#2-Cd(1)-N(1)	89.89(9)	O(1)-Cd(1)-N(1)	90.11(9)
O(1)#2-Cd(1)-N(1)#2	90.11(9)	O(1)-Cd(1)-N(1)#2	89.89(9)	N(1)-Cd(1)-N(1)#2	180.0
O(1)#2-Cd(1)-O(2)	124.89(8)	O(1)-Cd(1)-O(2)	55.11(8)	N(1)-Cd(1)-O(2)	89.93(9)
N(1)#2-Cd(1)-O(2)	90.07(9)	O(1)#2-Cd(1)-O(2)#2	55.11(8)	O(1)-Cd(1)-O(2)#2	124.89(8)
N(1)-Cd(1)-O(2)#2	90.07(9)				
**Complex 2**					
Bond	Dist.	Bond	Dist.	Bond	Dist.
N(4)-Cd(1)#1	2.317(18)	O(3)-Cd(1)#2	2.381(17)	Cd(1)-N(4)#3	2.317(18)
Cd(1)-N(1)	2.324(18)	Cd(1)-O(2)	2.364(16)	Cd(1)-O(1)#4	2.366(16)
Cd(1)-O(3)#5	2.381(17)	Cd(1)-O(4)#5	2.393(17)	Cd(1)-O(1)	2.522(16)
Angle	(°)	Angle	(°)	Angle	(°)
N(4)#3-Cd(1)-N(1)	176.5(7)	N(4)#3-Cd(1)-O(2)	94.7(6)	N(1)-Cd(1)-O(2)	87.4(6)
N(4)#3-Cd(1)-O(1)#4	90.8(6)	N(1)-Cd(1)-O(1)#4	85.7(6)	O(2)-Cd(1)-O(1)#4	125.9(5)
N(4)#3-Cd(1)-O(3)#5	86.5(7)	N(1)-Cd(1)-O(3)#5	93.7(7)	O(2)-Cd(1)-O(3)#5	140.2(6)
O(1)#4-Cd(1)-O(3)#5	93.7(6)	N(4)#3-Cd(1)-O(4)#5	90.3(7)	N(1)-Cd(1)-O(4)#5	92.7(7)
O(2)-Cd(1)-O(4)#5	85.7(6)	O(1)#4-Cd(1)-O(4)#5	148.2(6)	O(3)#5-Cd(1)-O(4)#5	54.6(6)
N(4)#3-Cd(1)-O(1)	92.9(6)	N(1)-Cd(1)-O(1)	86.1(6)	O(2)-Cd(1)-O(1)	53.3(5)
O(1)#4-Cd(1)-O(1)	72.7(6)	O(3)#5-Cd(1)-O(1)	166.5(6)	O(4)#5-Cd(1)-O(1)	139.0(5)
**Complex 3**					
Bond	Dist.	Bond	Dist.	Bond	Dist.
Cd(1)-O(3)	2.253(5)	Cd(1)-N(5)	2.319(6)	Cd(1)-O(8)	2.346(9)
Cd(2)-O(2)#2	2.255(4)	Cd(2)-N(2)	2.303(5)	Cd(2)-O(1)	2.309(4)
Cd(2)-N(1)	2.325(6)	Cd(2)-O(6)#3	2.367(5)	Cd(2)-O(5)#3	2.398(5)
Angle	(°)	Angle	(°)	Angle	(°)
O(3)#1-Cd(1)-O(3)	180.00(11)	O(3)#1-Cd(1)-N(5)#1	88.7(2)	O(3)-Cd(1)-N(5)#1	91.3(2)
O(3)#1-Cd(1)-N(5)	91.3(2)	O(3)-Cd(1)-N(5)	88.7(2)	N(5)#1-Cd(1)-N(5)	180.0(3)
O(3)#1-Cd(1)-O(8)#1	82.2(3)	O(3)-Cd(1)-O(8)#1	97.8(3)	N(5)#1-Cd(1)-O(8)#1	90.0(4)
N(5)-Cd(1)-O(8)#1	90.0(4)	O(3)#1-Cd(1)-O(8)	97.8(3)	O(3)-Cd(1)-O(8)	82.2(3)
N(5)#1-Cd(1)-O(8)	90.0(4)	N(5)-Cd(1)-O(8)	90.0(4)	O(8)#1-Cd(1)-O(8)	180.000(1)
O(2)#2-Cd(2)-N(2)	95.93(19)	O(2)#2-Cd(2)-O(1)	122.65(17)	N(2)-Cd(2)-O(1)	87.30(18)
O(2)#2-Cd(2)-N(1)	88.3(2)	N(2)-Cd(2)-N(1)	71.0(2)	O(1)-Cd(2)-N(1)	83.8(2)
O(2)#2-Cd(2)-O(6)#3	146.95(17)	N(2)-Cd(2)-O(6)#3	90.31(18)	O(1)-Cd(2)-O(6)#3	89.97(17)
N(1)-Cd(2)-O(6)#3	90.4(2)	O(2)#2-Cd(2)-O(5)#3	91.94(17)	N(2)-Cd(2)-O(5)#3	99.5(2)
O(1)-Cd(2)-O(5)#3	144.07(16)	N(1)-Cd(2)-O(5)#3	88.3(2)	O(6)#3-Cd(2)-O(5)#3	55.02(16)

Symmetry transformations used to generate equivalent atoms: **1**: #2 −x − 1, −y + 1, −z + 1. **2**: #1 x + 1, y + 1, z; #2 −x, y + 1/2, −z + 3/2; #3 x − 1, y − 1, z; #4 − x, −y + 1, −z + 1; #5 −x, y − 1/2, −z + 3/2. **3**: #1 −x + 1, −y + 1, −z + 1 #2 −x + 2, −y + 1, −z; #3 −x + 1, −y + 1, −z; #4 −x + 1, −y + 2, −z; #5 x, y − 1, z − 1, #6 x, y + 1, z + 1.
